# Pear Recognition in an Orchard from 3D Stereo Camera Datasets to Develop a Fruit Picking Mechanism Using Mask R-CNN

**DOI:** 10.3390/s22114187

**Published:** 2022-05-31

**Authors:** Siyu Pan, Tofael Ahamed

**Affiliations:** 1 Graduate School of Science and Technology, University of Tsukuba, 1-1-1 Tennodai, Tsukuba 305-8577, Japan; s2121091@u.tsukuba.ac.jp; 2 Faculty of Life and Environmental Sciences, University of Tsukuba, 1-1-1 Tennodai, Tsukuba 305-8577, Japan

**Keywords:** Mask R-CNN, 3D stereo camera, pear detection

## Abstract

In orchard fruit picking systems for pears, the challenge is to identify the full shape of the soft fruit to avoid injuries while using robotic or automatic picking systems. Advancements in computer vision have brought the potential to train for different shapes and sizes of fruit using deep learning algorithms. In this research, a fruit recognition method for robotic systems was developed to identify pears in a complex orchard environment using a 3D stereo camera combined with Mask Region-Convolutional Neural Networks (Mask R-CNN) deep learning technology to obtain targets. This experiment used 9054 RGBA original images (3018 original images and 6036 augmented images) to create a dataset divided into a training, validation, and testing sets. Furthermore, we collected the dataset under different lighting conditions at different times which were high-light (9–10 am) and low-light (6–7 pm) conditions at JST, Tokyo Time, August 2021 (summertime) to prepare training, validation, and test datasets at a ratio of 6:3:1. All the images were taken by a 3D stereo camera which included PERFORMANCE, QUALITY, and ULTRA models. We used the PERFORMANCE model to capture images to make the datasets; the camera on the left generated depth images and the camera on the right generated the original images. In this research, we also compared the performance of different types with the R-CNN model (Mask R-CNN and Faster R-CNN); the mean Average Precisions (mAP) of Mask R-CNN and Faster R-CNN were compared in the same datasets with the same ratio. Each epoch in Mask R-CNN was set at 500 steps with total 80 epochs. And Faster R-CNN was set at 40,000 steps for training. For the recognition of pears, the Mask R-CNN, had the mAPs of 95.22% for validation set and 99.45% was observed for the testing set. On the other hand, mAPs were observed 87.9% in the validation set and 87.52% in the testing set using Faster R-CNN. The different models using the same dataset had differences in performance in gathering clustered pears and individual pear situations. Mask R-CNN outperformed Faster R-CNN when the pears are densely clustered at the complex orchard. Therefore, the 3D stereo camera-based dataset combined with the Mask R-CNN vision algorithm had high accuracy in detecting the individual pears from gathered pears in a complex orchard environment.

## 1. Introduction

Modern fruit harvesting is mainly conducted by human labor and is roughly the same in different regions of the world. However, it requires human involvement and thus, complexity and labor hiring from overseas. The globalization of the COVID-19 pandemic and its economic impact has wreaked havoc on all economies around the world, pushing many into recession and possibly even economic depression [1]. Furthermore, the aging and availability of labor are concerns. Among common fruits, the pear stands out as an essential fruit type for daily life. For example, the Japanese pear (such as *Pyrus pyrifolia* Nakai) is one of the most widely grown fruit trees in Japan and has been used throughout the country’s history [2]. Regardless of the harvest season, due to the need for a large number of laborers for picking and a shortage of labor, the cost of pear picking has gradually increased.

The world labor force is predicted to decline by approximately 30% between 2017 and 2030 [3]. With the development of agricultural machinery, modern agricultural technology has gradually evolved from manual planting and picking to full automation and intelligence. Since the 1990s, with the development of computers and information technology, artificial intelligence and machine vision in agricultural machinery have become more effective and popular [4]. Since most agricultural work involves repetitive content operations, one of the most popular agricultural robots is the picking robot. Over time, most countries in the world have developed intelligent picking robots through different methods and techniques to load and unload agricultural products and detect fruit and positioning issues [5]. Therefore, for relatively delicate and soft fruits such as pears, the use of picking robots can greatly increase productivity. However, in recent studies, object detection in picking robots was reported to cause injuries due to grasping or using shear to detach the fruit from the branch [6]. The successful picking of soft pears depends on the recognition of the shape of the pears to understand the curved surface of the fruit. In classical image processing, it is challenging to recognize fruits, as shapes and sizes vary in orchards. In addition, illumination is a concern in dense canopies. Variability occurs in the detection of pears due to their size, shape, and illumination. Therefore, a large number of training datasets including size, shape, and illumination variabilities are needed to address the challenges of pear detection in complex orchard environments.

Deep learning has become a potential method to overcome the limitation of conventional segmentation in image analysis. It is one of the subfields of machine learning and has now developed a variety of different architectures [7]. Self-Organizing Feature Map (SOFM) is the ability of the discussed neural network to determine the degree of similarity that occurs between classes. It is also a method that belongs to deep learning. Among other things, SOFM networks can be used as detectors that indicate the emergence of a widely understood novelty. Such a network can also look for similarities between known data and noisy data. [8] Additionally, deep learning includes artificial neural networks (ANNs) [9] and neural networks extracted by convolutional neural networks (CNNs) by fully connected layers (FCNs), where CNNs preserve the spatial relationships between pixels by learning internal features using small pictures of the input data [10].

Intelligent robot vision processing of target plants has become an indispensable step in agricultural intelligence and many excellent target detection methods are now widely used in the development of agricultural robots as target detection continues to develop. The first types included Fast R-CNN [11] and Faster R-CNN [12], which have roughly the same principle of selecting the region of interest by region feature network (RPN) [13] and then transmitting to the head layer to generate the edges as well as the species. With the demand for accuracy in target detection, Mask R-CNN [13] was introduced, which adds FPN [13] to the backbone layer based on Faster R-CNN and adds a new branch in the head layer to generate more accurate masks. The second type is You Only Look Once (YOLO) [14], which focuses on the detection of targets; all detection results are lower than the above models but faster than the above models. However, due to the demand for accuracy in target detection, the Mask R-CNN detection speed is slower than that of other detection models [15].

Some identification techniques identify by evaluating, extracting, and recognizing color, because, in the food industry, color is an identifier used by producers and processing engineers as well as consumers and is the most direct way of identification [16]. Therefore, color extraction is also widely used in identification technology. Boniecki et al. 2010 analyzed the classification ability of Kohonen-type neural models learned using “unsupervised” methods. Classification of three selected apple varieties frequently found in Polish orchards was carried out. The neural classification was based on information encoded in the form of a set of digital images of apples and dried carrots. Representation in the form of a palette of the main colors occurring in fruits and dried vegetables and selected shape coefficients were used as a basis for the classification [17].

However, it is not enough for deep learning. Most of the deep learning methods were limited to RGB images which have a limitation of depth information. In a recent study, a thermal camera was used to detect tree trunks in a complex orchard. However, in comparing a tree trunk to some fruit, there is more complexity in detecting and measuring the distance for picking information [18]. A 3D stereo camera has further advantages in addition to conventional camera sensors. The 3D stereo camera mimics and imitates the human eye imaging principle. With the powerful visual system of the human eye, the perception of the third dimension (depth) is derived from the difference between the image formed by the left eye and the right eye. Because of this difference, the human eye visual system introduces the third dimension (depth), and the 3D stereo camera receives biological inspiration to detect the depth information of an object by extracting three dimensions of information from the digital image and using it for 3D reconstruction. In addition, the camera perceives the depth of objects in the range of 1 to 20 m at 100 FPS [19]. By detecting complex situations in orchards with a 3D stereo camera combined with Mask R-CNN vision algorithms, specified fruits can be detected.

Mask R-CNN is conceptually simple and was proposed by Kaiming et al (2017). It is a flexible, pass-through object instance segmentation framework. This method can efficiently detect objects in images while generating high-quality segmentation masks for each instance. Mask R-CNN was also used for instance segmentation of detected objects and the evaluation of human poses [13]. Several studies have shown that Mask R-CNN can be used for the detection of some fruits. Jia et al (2020) used a series of apple images with a size of 6000 × 4000 -pixel resolution under natural light using a Canon camera for cloudy and sunny weather conditions [20]. Yu et al (2019) proposed a Mask R-CNN-based algorithm to detect and quantify wild strawberries, and the fruit detection results of 100 test images showed an average detection accuracy of 95.78% and a recall rate of 95.41% [21]. All of the above results showed that Mask R-CNN can be used for instance segmentation. In the above study, RGB images were used, which did not cover the depth information of the distance. However, Mask R-CNN with a 3D stereo camera can be further used for complex canopy and the weight files produced by the dataset produced by the common dataset. However, the 3D stereo camera has problems such as recognition errors and difficulty in obtaining depth information when detecting in real time. If the additional function has the depth information of the garden, then masking in terms of shape and size is still possible.

Mask R-CNN extended the object detection framework of Faster R-CNN by adding an additional branch at the end of the model, thus achieving instance segmentation for each output suggestion frame using a fully connected layer [22]. Unlike ROI-Pooling of Faster R-CNN, ROI-Pooling inputs an image and multiple regions of interest (ROIs) into a feature map of fixed size, which was then mapped to a feature vector by a fully connected network (FCN) [11]. However, ROI-Align in Mask R-CNN canceled the quantization of ROI-Pooling twice and retained the decimals, and then used bilinear interpolation [23] to obtain the image values on pixel points with floating-point coordinates. This was because although the quantization did not affect the classification in the work, it had a significant negative impact on predicting the exact mask for pears in the orchard [13].

However, the complexity of orchards causes difficulty in detection, such as the presence of leaf shading, overlapping fruits, insufficient light, interruption of light due to nets over the canopy, and more shadows in orchards, which affect the detection results. Faster R-CNN was used to detect peppers, melons, and apples using multiple vision sensors [24], and although high detection accuracy was achieved, the detection of overlapping fruits was greatly reduced. Mask R-CNN has the potential to help overcome problems with size, shape, and illumination. Since the Mask R-CNN uses instance segmentation, it can over detect different individuals of the same species, so overlapping parts of the fruit can also be detected precisely and variability in shape can be adjusted, thus improving the accuracy of detection. Therefore, the purpose of this research is to develop a pear recognition system using instance segmentation based on a Mask RCNN from 3D camera datasets. The expected recognition of pears can be implemented as a fruit picking mechanism with fewer injuries to the surface with the recent advancements of manipulators and robots.

## 2. Materials and Methods

### 2.1. Field Data Collection

In this study, a 3D stereo camera named ZED (Stereolabs Inc., San Francisco, CA, USA) was used to collect 3018 (4-channel) original pictures from the T-PIRC (36°07′04″ N, 140°05′45″ E) on a sunny day. The video shot with the ZED camera simulated the movement of the trajectory of the manipulator, and the observation distance from the pear was less than 50 cm. The video was trimmed into a frame-by-frame binocular image through the ZED camera protocol. The right side of the camera was showed the images in depth images, and the left side was 4-channel RGBA images.

**Figure 1:**
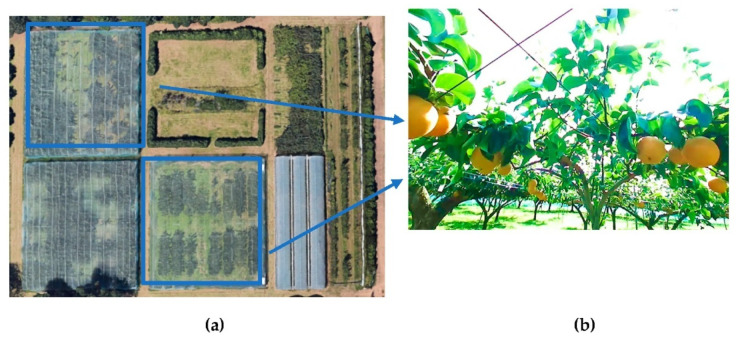
Aerial view of orchards for data collection located at the Tsukuba-Plant Innovation Research Center (T-PIRC), University of Tsukuba, Tsukuba, Ibaraki**.** (**a**) satellite view of Tsukuba-Plant Innovation Research Center (T-PIRC); (**b**) the view of pear orchard in T-PIRC.

Considering the influences of different light intensities in the natural environment and the camera parameters, in this research, four videos were taken at 9–10 a.m. and four video were taken at 6–7 p.m. from Tsukuba-Plant Innovation Research Center (T-PIRC). The videos were taken during pears were at the fruit stage grown in the orchards covered with nets (Figure 1a,b). The total number of original images used was 3018 in the training, validation, and testing process. Among these datasets, there were 1818 images used for training, 900 images for validation, and 300 images for testing. As mentioned before, that we collected the data from different times with different light intensities included high light and low light conditions (Table 1).

### 2.2. Instance Segmentation

**Figure 2:**
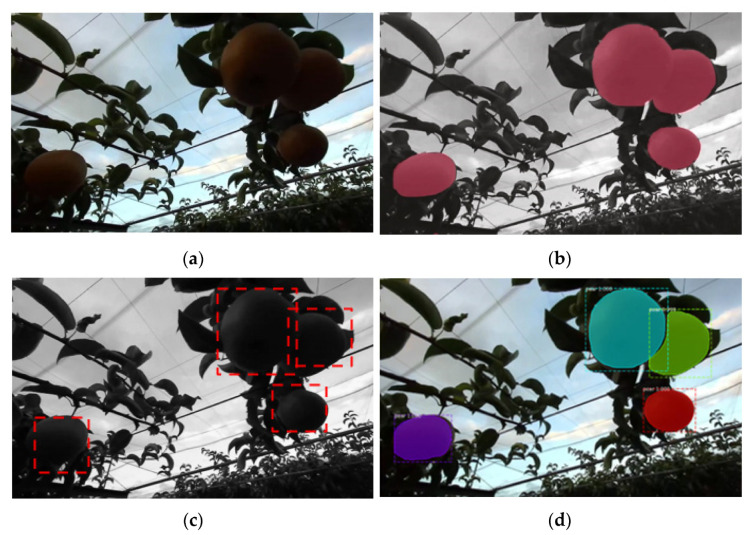
Different segmentation in pear detection using 3D camera datasets, (**a**) original image; (**b**) semantic segmentation; (**c**) object detection and (**d**) instance segmentation.

Image segmentation techniques consisted of object detection, semantic segmentation, and instance segmentation. Object detection solved the problem of identifying the content and location of images. Semantic segmentation was used to label each object with classes. However, instance segmentation was a combination of object detection with boundaries and semantic segmentation with classes (Figure 2). In the case of instance segmentation, the object pear fruit was recognized as individual pears inside the same class compared to semantic segmentation. Many instance segmentation methods were based on segmentation proposals [13]. Deep-Masks [24] proposes segmentation candidates followed by Fast R-CNN for classification. These methods were very slow and inaccurate. Mask R-CNN performs parallel prediction based on the Mask and labels, making its instance segmentation method simpler and faster [13].

### 2.3. Mask R-CNN

**Figure 3:**
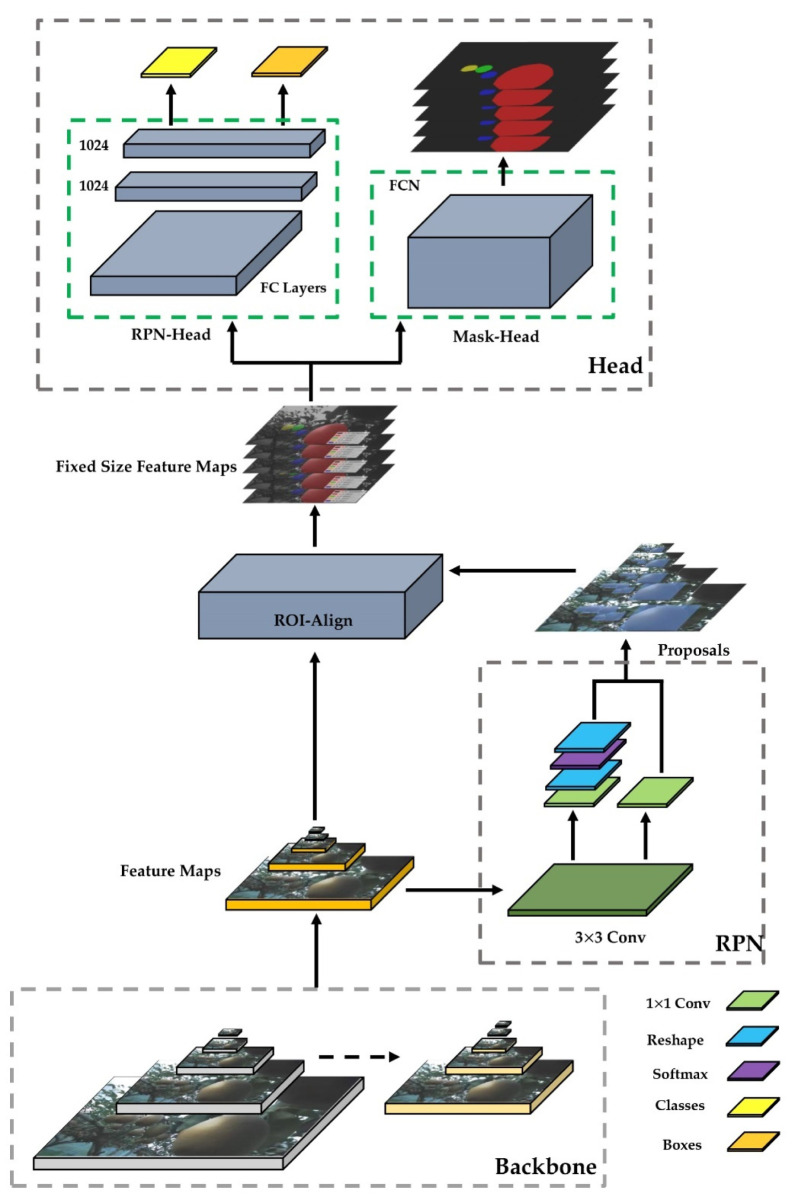
Mask R-CNN structure for pear quantity in orchards from 3D camera datasets. The original images enter the backbone network for selection and screening to get the feature maps. Then, the foreground and background are extracted in the RPN network, enter the ROI-Align network for standardization, and finally enter the head network to generate classes, boxes, and masks for pear detection.

In the MASK R-CNN process, three main parts were followed: first, the backbone network extracts feature maps from the input image; second, the feature map outputs from the backbone network were sent to the Region Proposal Network (RPN) [12] to generate regions of interest (ROIs); third, the ROI maps were output from the RPN, mapped to the shared feature maps to extract the corresponding target features, and then output to the FC and full convolutional networks (FCN) for target classification and instance segmentation (Figure 3). This process generated classification scores, bounding boxes, and segmentation masks. With the evidence of the presented research, Mask R-CNN was used to detect the fruit.

### 2.4. ZED AI Stereo Camera

This research used a stereo camera as the main camera for data collection. The stereo camera was an integrated binocular camera that used advanced sensing technology based on stereo vision to provide video acquisition, depth information, real-time location information, and other technologies. It has been applied to target reconstruction, position acquisition, and other fields [25]. A stereo camera was used to obtain the distance of the pear through its depth functions, such as a 3D point cloud map. Therefore, the distance between the target fruit with the mask to the camera is measured and transmitted to the upper computer. Then, to realize the simulated grasping of the robot, Mask-RCNN was used for apple pickings and implemented in the intelligent platform.

### 2.5. Data Preparation

#### 2.5.1. Deep Learning Environment

This experiment used a processor 11th Gen Intel(R) Core (TM) i7-11700F @2.50 GHz(16CPUs), ~2.5 GHz, 16,384 MB RAM, and Nvidia GeForce RTX 3060 GPU with Windows® 10 home edition™, CUDA 10.0, cuDNN 7.4, and Visual Studio™ 2019 as the training base. The environment configuration was created based on Mask R-CNN environment under anaconda, where the TensorFlow version was used in TensorFlow-gpu2.14.0, Keras 2.6.0, and Python 3.6.

#### 2.5.2. Video to Image Conversion

The videos were taken with a stereo camera and images were converted to a specific format. Since the stereo camera came with its own shooting software, ZED Explorer™, which could shoot videos in three modes (ULTRA, PERFORMANCE, and QUALITY), the PERFORMANCE mode was chosen for this experiment, shooting a number of videos in HD720 stored in SVO format. The ZED protocol was used to trim the images to PNG format, and the image resolution was 1280 × 720.

#### 2.5.3. Image Annotation

LabelMe^®^ was used as the image annotation tool for semantic segmentation written in JavaScript for online labeling [26]. The difference from LabelImg^®^ was that the target was plotted in detail, and then a target mask was generated in LabelMe. LabelMe labeled all targets under the software interface, and different classes were named as different label tags and different entities of the same class were named at once in order.

### 2.6. Data Splitting

The dataset consisted of 3018 images taken at different times of the day. The Mask R-CNN dataset was divided into training, validation, and testing sets, with the ratio set at 6:3:1.

In earlier experiments, three sets of videos with 1080 P resolution and .avi format were taken with an iPhone™ 11 mobile phone. We observed that the training set taken with the ZED camera made it difficult to test the videos and images taken with the mobile phone. This was due to the different camera calibration modules used on the stereo and the mobile phone camera as well as the different apertures and light transmission of the cameras making accurate identification difficult. This was why all of the experiments were conducted using stereo cameras to produce the dataset and test set. Second, as in the video taken by the stereo camera, different colors of pears existed in different shadows, so the individual pears in the dataset also showed three colors: bright yellow, yellow, and dark yellow. In this study, all colors of pears were calibrated and placed in the training set and the validation set to achieve adaptation to each angle and each color of pears.

Additionally, due to the homogenization of the original dataset, the shape of the pears and leaves tended to be similar under the dark light condition. Therefore, we decided to perform data augmentation on this dataset. Since the shape of the pear is similar to a sphere, and the shape of the leaf was irregular, we flipped and rotated each image of the original dataset so that the pear still tended to be spherical at different angles, but the leaves presented different shapes at different angles. The data set was expanded to 9054 images with the training set having 5054 images, the validation set having 2700, and the testing set having 900 at the ratio of 6:3:1 by data augmentation. We also rotated each image of the original dataset by 30° and flipped each image by 180° with the same method.

### 2.7. Training Process of Mask R-CNN

#### 2.7.1. Feature Extraction (Backbone: ResNet101 + FPN)

A deep convolutional network referred to a network with different depths that could be constructed by constructing different weights. This was widely used in image feature extraction. However, with further deepening of the convolution network, the more convolution layers, the higher the corresponding training error. For the original network, simply increasing the depth led to gradient dispersion or exploding gradients. To address this issue, we represented this layer as an input-based learned residual function. Experiments showed that residual networks were easier to optimize and improved accuracy by adding considerable depth [27].

**Figure 4:**
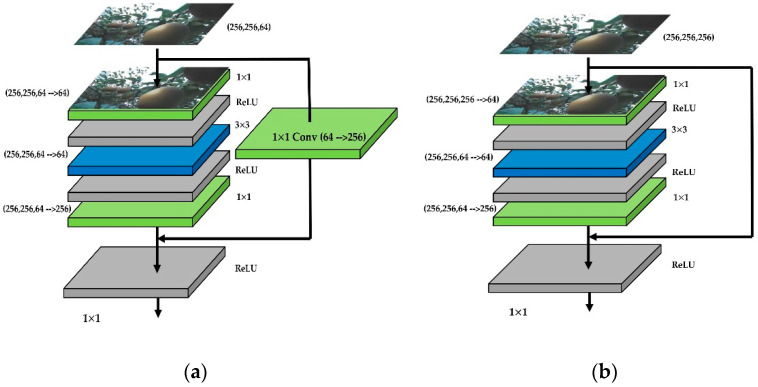
The inner structure of ResNet101 as an example of second layers (C2): (**a**) is conv block and (**b**) is identity block. The images which were inputted into the ResNet have changed the channels. Conv block is the first stage of each layer, and the identity blocks and conv blocks were combined to the ResNet.

Residual networks (ResNet) were widely cited in the backbone networks of Faster R-CNN and Mask R-CNN, with ResNet50 and ResNet101 being the most common for Mask R-CNN. The Mask R-CNN using ResNet101 outperforms all previous basic variants of the state-of-the-art model [13], including the single-model variant of G-RMI [28] (Figure 4).

These figures showed the different residual modules in a stage. The basic structure of these two modules met the standard residual structure. The difference between the convolutional block and identity block was that the convolutional block had a 1 × 1 conv layer. The shortcut of the convolutional block needed to go through a 1 × 1 conv for converting the number of channels. The identity block was directly connected to the upper output level. Therefore, ResNet101 was chosen as the backbone for the Mask R-CNN in this research.

**Figure 5:**
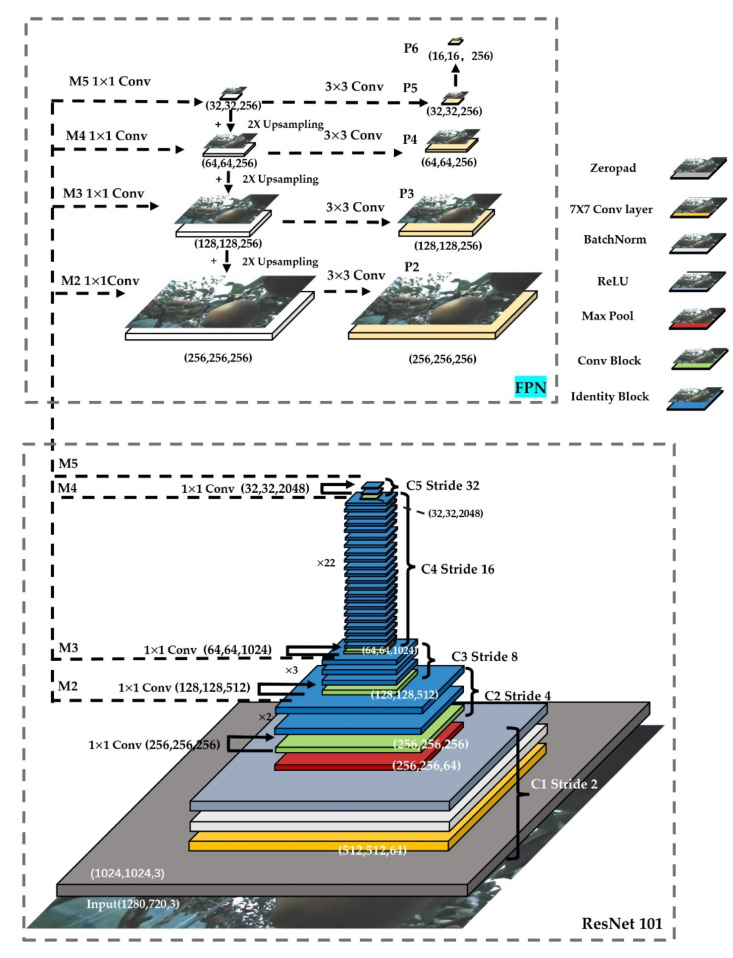
ResNet101 + FPN for pear quantity recognition.

The feature pyramid network (FPN) [29], as an elaborate multiscale detection method, had a structure consisting of three parts: bottom-up, top-down, and lateral connections. This structure allowed the features of each layer to be fused so that they had both strong semantic and strong spatial information. Feature extraction using the ResNet-FPN backbone for Mask R-CNN showed a great improvement in accuracy and speed [13]. The structure of Mask-RCNN feature extraction was based on ResNet 101 and FPN (Figure 5).

Top-down: no difference from the traditional feature extraction process, ResNet was used as the skeleton network and then divided into five stages according to the size of the feature map. These were named Stage 1, Stage 2, Stage 3, Stage 4, and Stage 5. In the convolution process, Conv2, Conv3, Conv4, and Conv5 are defined as C2, C3, C4, and C5. Next, the stage passes through the FPN on the right side from top to bottom and from left to right. Sampling starts from the last layer and samples to the nearest upper layer. The results of the previous layer were connected horizontally by a layer conv 1 × 1, which was used to reduce its channels, and the results of all sampled layers were set to the same channels, fixed at 256. From there, it was processed through a layer of conv 3 × 3 to eliminate the aliasing effect. P2, P3, P4, P5, and P6 from FPN were used as RPN inputs, and P2, P3, P4, and P5 were used as subsequent Mask R-CNN inputs [28].

#### 2.7.2. Region Proposal Network (RPN)

**Figure 6:**
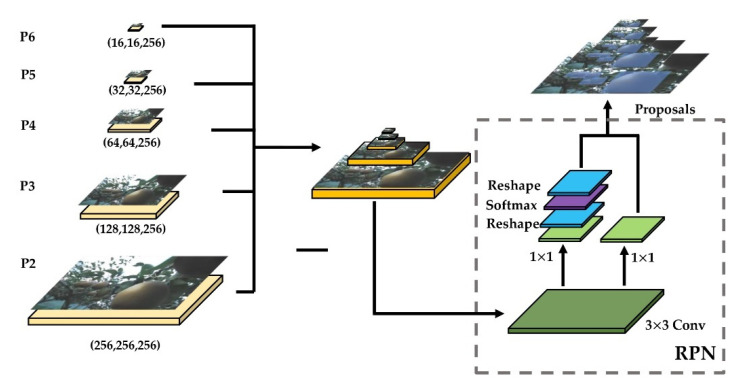
RPN in Mask R-CNN for extracting proposals of original pear images.

RPN, as a fully convolutional network (FCN) [30], was specifically targeted for the task of generating detection suggestions and extracting candidate frames [11]. Based on P2, P3, P4, P5, and P6 obtained in 2.6.1, a series of anchors were generated in the RPN. Taking the P6 layer as an example, the feature map size of the P6 layer was (16, 16, and 256), and its step size relative to the original map was 64 so that each pixel point on P6 was generated with three transformations of anchors with aspect ratio {0.5, 1, 2}. There were a total of 1200 generated anchors (16 × 16 × 3 = 768), and by analogy, P2 (256 × 256 × 3 = 196,608), P3 (128 × 128 × 3 = 49,152), P4 (64 × 64 × 3 = 12,288), and P5 (32 × 32 × 3 = 3072); from P2 to P5, a total of 261,888 anchors were generated on the original image (Figure 6).

**Figure 7:**
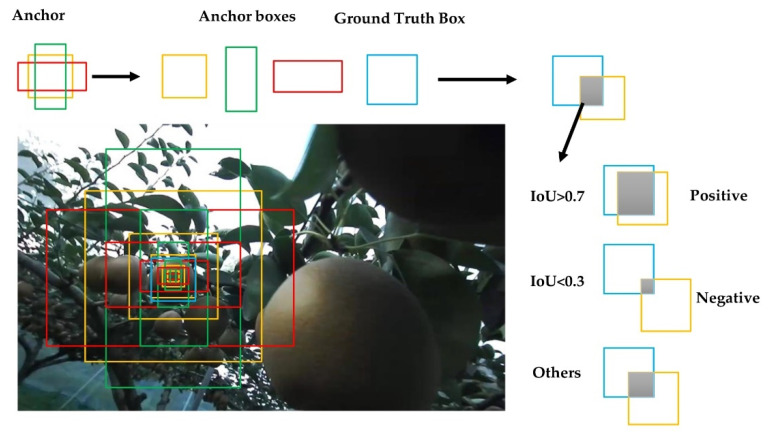
Generation for IoU by comparing anchor boxes with ground truth boxes. If IoU > 0.7, then label = 1 positive; if IoU < 0.3, then label = −1 negative; others, label = 0.

The positive and negative classes for network training were established by the generated anchors and 256 were selected according to intersection-over-union (IoU) [12] for training the RPN, of which, 128 positive samples (foreground) and 128 negative samples (background) were guaranteed. This step was performed to calibrate the anchor box, in addition to calculating the offset between the anchor box and the ground truth (Figure 7).

The IoU represented the overlap of the two bounding boxes, where the ground truth and the offset of the anchor box can be expressed as

(1) $${∆}_{X}^{*}\;=\;\left(x^{*}\;-\;x_{a}\right)/w_{a},{∆}_{Y}^{*}\;=\;\left(y^{*}\;-\;y_{a}\right)/h_{a}$$

(2) $${∆}_{W}^{*}\;=\;\log\left(\frac{w^{*}}{w_{a}}\right),{∆}_{H}^{*}\;=\;\log\left(\frac{h^{*}}{h_{a}}\right).\;$$

where $x_{a}$, $y_{a}$ represented the coordinate value of the center point of the anchor box; $w_{a}$, $h_{a}$ represented the width and height of the anchor box; $x^{*}$, $y^{*}$ represented the coordinate value of the center point of the ground truth; and $w^{*}$, $h^{*}$ represented the coordinate value of the width and height of the ground truth.

Next, we entered the regression and classification of the RPN which classified each layer of the anchor into the foreground and background and the four displacement quantities of regression. For example, in the P6 layer, the size of the feature map was 16 × 16, there were 16 × 16 × 3 anchors, and the probability of each anchor as the foreground and background was calculated separately. The array was (16 × 16 × 3, 2), and the regression of offset was (16 × 16 × 3, 4). The same operation was performed from P2 to P6, and the classification of (261,888 and 2) was obtained in the total information and regression information of (261,888 and 4).

The scores (probabilities) corresponding to the 256 positive and negative samples were found from (261,888 and 2); as a result, 256 positive and negative samples were obtained. The Softmax cross-entropy loss values were calculated using the scores and the label values of the positive and negative samples, which caused RPN to initially extract foreground anchors as candidate regions using anchors and the Softmax function.

The offset corresponding to the index where the 128 positive samples were located from the (261,888 and 4) regression array was found and this offset was used with the offset calculated between the positive samples and the real frame to calculate the loss value to regress the proposals from the positive samples.

#### 2.7.3. ROIs and ROI-Align

In this research, the ROI parameter was set for taking a certain amount from 261,888 anchors as the ROI during training; this parameter was set to 2000. Therefore, 2000 anchors from 261,888 anchors were taken as the ROI needed in the next stage.

Our method ranked the scores of the positive samples obtained in the previous stage of RPN from highest to the lowest, removed the top 2000 anchors with the highest scores, and accumulated the more accurate box coordinates by regression of the offset of RPN. Finally, a nonmaximal suppression (NMS) [31] was performed on these 2000 anchors to eliminate duplicate boxes. Finally, 2000 matching ROIs were selected.

Since NMS processing was performed on the ROIs after the proposal, some of the layers with less than 2000 ROIs had supplemental 0 processing, so the 2000 ROIs obtained needed to be eliminated. Eliminating the ROIs filled with 0’s and eliminating all the boxes with multiple objects in the ground truth at the same time, the IoU value of each ROI was calculated. Then, we calculated the IoU value of each ROI and ground truth, obtained 400 ROIs with positive and negative samples of 1:3, and finally returned its 400 samples, its displacement offset, and 400 masks.

ROI-Align of the standardized ROIs was used to obtain the final required feature maps. ROI-Align was a unique part of Mask R-CNN. Unlike ROI-Pooling in Faster R-CNN, ROI-Pooling was a standardized operation used to extract a small feature map from each ROI, and this feature had a fixed spatial range. This paper used 7 × 7 feature maps as fixed-size feature maps. First, the ROI of the floating-point number was quantized into a feature map of standard size, and the quantized ROI was quantized again to obtain an N × N integer feature map. Furthermore, for an image whose original size was 1280 × 720, after eight samplings and resizing, the size of the obtained feature map was 160 × 160. Assuming that there was a 113 × 113 region proposal, the size of the feature map was 14.125 × 14.125 (113/8). At this time, after the first quantization, the region proposal size on the feature map was 14 × 14. Assuming that it eventually became a 7 × 7 fixed-size feature map, the feature map needed to be divided into 49 regions, and the size of each region was 14/7 = 2. At this time, the second quantization was performed, and the final small area size was 2 × 2. Finally, the maximum pixel quality was selected in each small 3 × 3 area to form a 7 × 7 fixed-size feature map.

**Figure 8:**
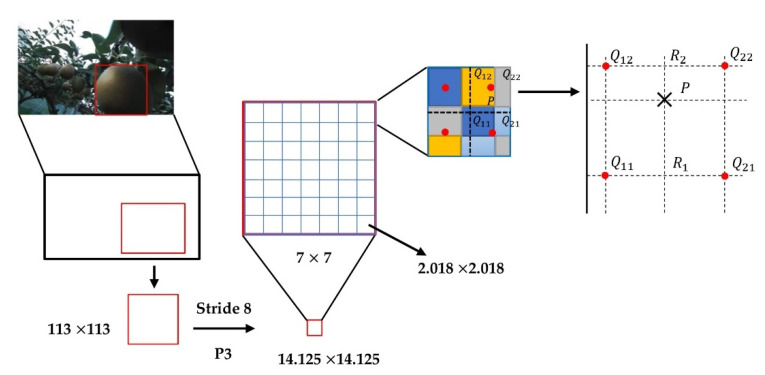
Bilinear interpolation in ROI-Align was used to obtain fixed feature maps for pear recognition. P represents pixel coordinates that ROI-Align wanted to obtain after bilinear interpolation. Q11, Q12, Q22, and Q21 represent the four coordinates of known pixel points around point P.

Although ROI -Pooling did not change the categories in the data in the two quantization while it generated the mask, omitting the very small floating-point number also affected the area and size of the mask. Mask R-CNN canceled the two in ROI-Pooling-quantization operations and preserved floating-point numbers [13]. For example, images were taken at a size of 1280 × 720. After eight samplings, the size of the feature map (8 × 8) was obtained. Another 113 × 113 region proposal was also assumed to be mapped to the feature map. The size then changed to 14.125 × 14.125. The operation at this time was different from the above ROI-Pooling. The quantization operation was directly canceled, and the floating-point number was reserved. Assuming that the final required transformation was a 7 × 7 feature map, 49 small areas needed to be planned in the feature map with a feature map of 14.125 × 14.125. Since the size of each small area was changed to 14.125/7 = 2.018, the final minimum area was 2.018 × 2.018. Assuming that the number of sampling points was four, bilinear interpolation was used for calculation so that the pixel values of the four points could be obtained. The quantization operation was eliminated, and the errors were greatly reduced (Figure 8).

The process of ROI-Align simply described and calculated the edge lengths of each ROI but does not round them. Each ROI region was divided into K × K bins, and the size of each bin was not rounded. The value of each bin was obtained by bilinear interpolation of the four values of the most adjacent feature map. Max pooling was used to obtain a feature vector of fixed length [13]. Mask R-CNN uses bilinear interpolation, which is a useful operation that can be used to interpolate two-dimensional images to compare different pixel sizes or image spacings, which be used to remove ROI-Align in Mask R-CNN. Thus, the pixel values of four adjacent pixel points were obtained. For example, if the number of sampling points were four, this could be expressed as follows: for each small area, the average was divided into four points, the center point position was taken for each part, and the pixel value of the center point position at this time was bilinear. The interpolation method was used for calculation. Through this method, the pixel values of the four points were obtained, thereby eliminating the need for two quantization operations and generating the corresponding mask for pear recognition.

#### 2.7.4. Mask RCNN for Classification and Regression

The ROIs obtained after entering ROI-Align needed to pass the head architecture to perform category of classification, regression, and mask generation operations, which had two main branches. The upper branch indicated category classification and regression, and the lower branch generated the mask for each pear in each feature map.

**Figure 9:**
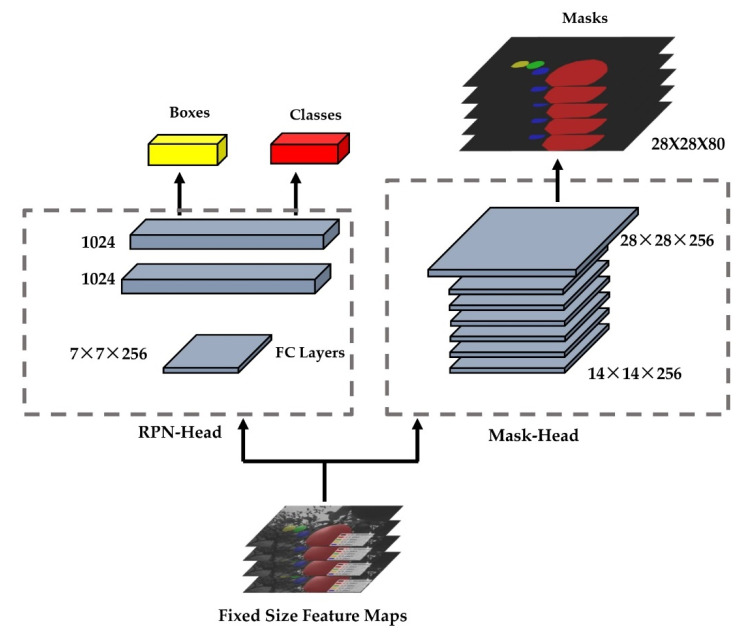
Flow diagram of feature maps to produce boxes, classes, and masks for each pear in each fixed-size feature map after ROI-Align.

After the ROI of the previous layer, a 7 × 7 × 256 feature map was generated, and another branch was aligned to a 14 × 14 × 256 feature map (Figure 9). Since the outputs of P2, P3, P4, P5, and P6 were all 256, the final channel was still 256 after ROI-Align.

The branch of left side in Mask R-CNN had the same principle of classifying targets and generating frames with Faster R-CNN. The Faster R-CNN also calculated which category each proposal belongs to and outputs the classification result through the fully connected layer. Additionally, the position offset of each proposal was obtained by bounding box regression which was used to return a more accurate target detection frame. The branch of right side was based on the Faster R-CNN, and the full convolutional layer branch was added to obtain a more accurate mask. The difference between FCN and CNN is that the final FCN was replaced by a convolutional layer. Unlike the classical CNN, which uses FCN after the convolution layer to obtain a fixed-length feature vector for classification (fully connected layer + Softmax output), FCN accepted an input image of arbitrary size and used deconvolution to sample the feature map of the last convolution layer to restore it to the same size as the output image. The feature map of a fixed size of the ROI region was generated by the ROI-Align operation. After four convolution operations, a 14 × 14 feature map was generated. Then, a 28 × 28 feature map was generated by up-sampling. Finally, a 28 × 28 feature map with a depth of 80 was generated by the deconvolution operation to obtain the exact mask.

#### 2.7.5. Loss Function

The loss functions of the Mask R-CNN were divided into two main parts. The first part was the RPN loss function, which was similar to the Faster R-CNN. The RPN loss function consisted of two parts: the classification loss ($L_{CLS}$) and the bounding box regression loss ($L_{BOX}$) [10].

(3) $$L\;=\;L_{\mathrm{RPN}}\;+\;L_{\mathrm{MASK}}$$

(4) $$L_{RPN}\;=\;L_{CLS}\;+\;L_{BOX}$$

(5) $$L(\{p_{i}\},\left\{u_{i}\right\})\;=\;\frac{1}{N_{cls}}\underset{i}{\sum }L_{cls}\left(p_{i},p_{i}^{*}\right)\;+\;\lambda \frac{1}{N_{reg}}\underset{i}{\sum }p_{i}^{*}L_{reg}\left(t_{i},t{\;}_{i}^{*}\right)\;$$

(6) $$L_{CLS}\;=\;\frac{1}{N_{cls}}\underset{i}{\sum }L_{cls}\left(p_{i},p_{i}^{*}\right)\;$$

*Ncls*: Since the anchor generated in the RPN stage was only used to classify foreground and background and was set to 256, the value of $\;N_{cls}$ is 256.

$p_{i}$ was the probability of predicting the target, $p_{i}^{*}\;$ was the Ground Truth (GT) label.

(7) $$p_{i}^{*}\;=\;\left\{\begin{array}{c} \;0\;,\;negative\;label\\ 1\;,\;positive\;label \end{array}\right.$$

$L_{cls}\left(p_{i},p_{i}^{*}\right)$ denotes two classes: the target and the nontarget logarithmic loss,

In which

(8) $$L_{cls}\left(p_{i},p_{i}^{*}\right)\;=\;-\log\left[p_{i}^{*}p_{i}\;+\;\left(1\;-\;p_{i}^{*}\right)\left(1\;-\;p_{i}\right)\right]\;$$

(9) $$L_{\mathrm{BOX}}\;=\;\lambda \frac{1}{N_{\mathrm{reg}}}\sum_{i}p_{i}^{*}L_{\mathrm{reg}}(t_{i},t_{i}^{*})$$

$N_{reg}$ was the size of the feature maps, and $\lambda \frac{1}{N_{\mathrm{reg}}}$ was used as the normalized weight to balance the classification loss and regression loss, which was taken as $\frac{1}{256}$ in the RPN training phase,

$t_{i}\;=\;\left\{t_{x},t_{y},t_{w},t_{h}\right\}$ was a vector that represents the offset used for prediction in the RPN training phase,

$t{\;}_{i}^{*}$ was the actual offset of the group truth corresponding to the positive anchor.

(10) $$L_{\mathrm{reg}}(t_{i},t_{i}^{*})\;=\;R(t_{i}\;-\;t_{i}^{*})$$

R was the $smooth_{L1}$ function

(11) $$smooth_{L1}\left(x\right)\;=\;\left\{\begin{array}{c} 0.5x^{2}\;if\;\left|x\right|<1\\ \left|x\right|\;-\;0.5\;otherwise \end{array}\right.\;$$

$L_{MASK}\;$ was the loss function resulting from adding the mask branch to the Mas R-CNN and had the functional expression

(12) $$L_{MASK}=L\left(p_{i},p_{i}^{*}t_{i}t_{i}^{*}s_{i}s_{i}^{*}\right)\;=\;\frac{1}{N_{cls}^{\prime }}\underset{i}{\sum }L_{cls}\left(p_{i},p_{i}^{*}\right)\;+\;\lambda^{\prime }\frac{1}{N_{reg}^{\prime }}\underset{i}{\sum }p_{i}^{*}L_{reg}\left(t_{i},t{\;}_{i}^{*}\right)\;+\;\gamma \frac{1}{N_{mask}}\underset{i}{\sum }L_{mask}\left(s_{i},s{\;}_{i}^{*}\right)$$

In this research, we conducted a discusion of the pseudo-code of Mask R-CNN for different datasets including training set (TRD), validation set (VAD), and testing set (TSD). We gave a brief logical explanation in a tabular format of the pseudocodes of the modified Mask R-CNN (Algorithms 1–4). The following table shows Pseudo-codes for Mask R-CNN and Faster R-CNN in training and predicting phases.


**Algorithm 1. Training Phase in Mask R-CNN**
1. **Inputs**:2.             Dataset_train: $TRD={\left\{Image_{i}\right\}}_{i=1}^{M}$,3.             Datset_val: $\mathrm{VAD}\;=\;{\left\{Image_{j}\right\}}_{j=1}^{N}$ ,where *M*, *N* are the number of images.4.               **if** mode = “training”5.                 Get object index in $Image_{i}$, $Image_{j}$6.                 Extraction from ResNet101 to FPN7.                 Anchor generation from
P2,P3,P4,P5,P68.                 BG and FG generation from  RPN via (Equations (1) and (2))9.                 Calculated the $L_{RPN}$ via (Equations (4)–(9))10.                 ROIs Generation from ROI−Align11.                 Masks, boxes, classes Generation from the Head12.                 Calculate the loss of the head layer $L_{MASK}$ via (Equation (12))13.                 Save_logs_weights(mask_rcnn_shapes.h5)14. **Return**:15.           $\;logs\_weights,\;L_{RPN},\;L_{MASK},\;L$



**Algorithm 2. Testing Phase in Mask R-CNN**
1. **Inputs**:2.             Dataset_Test: $TSD={\left\{Image_{i}\right\}}_{i=1}^{M}$, where *M* is the number of images.3.             GPU_COUNT = 14.             IMAGES_PER_GPU = 15.                **if** mode =” inference”6.                 Model.load_weights (mask_rcnn_shapes.h5)7.                 **For**
*i*
**in range** ($Image_{i}$):8.                    **Input**
Anchors
9.                    Generated rpn_ROIs
10.                  targer_ROIs=rpn_ROIs
11.                  Generated target_class_ids, target_bbox, target_mask
12.                  Created masks for detections13. **Return**:14.             $Image_{i}$, *masks*, *class_id*, *class_name*, *scores*15.          Visualize.display_instances $Image_{i}$



**Algorithm. 3 Training Phase in Faster R-CNN**
1. **Inputs**:2.             Dataset_train: $TRD={\left\{Image_{i}\right\}}_{i=1}^{M}$,3.             Datset_val: $VAD={\left\{Image_{j}\right\}}_{j=1}^{N}$, where M, N is the number of images.4.              **if** mode = “training”5.               Get object index in $Image_{i}$.6.               Extraction from
VGG16 (*Visual Geometry Group Network*)7.               Region proposals generation from RPN
8.               ROIs generation from
ROI−Pooling
9.               Classification from  the Head
10.                Calculated Loss
11.                Save_logs_weights12. **Return**:13.             *logs_weights*, *L_RPN_*


**Algorithm 4. Testing Phase in Faster R-CNN**
1. **Inputs**:2.             Dataset_Test: $TSD={\left\{Image_{i}\right\}}_{i=1}^{M}$, where *M* is the number of images.3.              if mode =” inference”4.               Model.load_weights5.               **For**
*i*
**in range** ($Image_{i}$):6.                **Input**
Anchors7.                Generated rpn_ROIs
8.                   targer_ROIs=rpn_ROIs
9.                Generated target_class_ids, target_bbox
10. **Return**:11.             $Image_{i}$, $Image_{i}$
12.           Visualize.display_instances
$Image_{i}$


#### 2.7.6. Model Metrics Function

The results of the model prediction values were classified into four categories: true positive (*TP*), indicating a positive sample detected correctly; false negative (*FN*), indicating a negative sample predicted incorrectly; true negative (*TN*), a negative sample predicted correctly; and false positive (*FP*), a positive sample predicted incorrectly.

**Figure 10:**
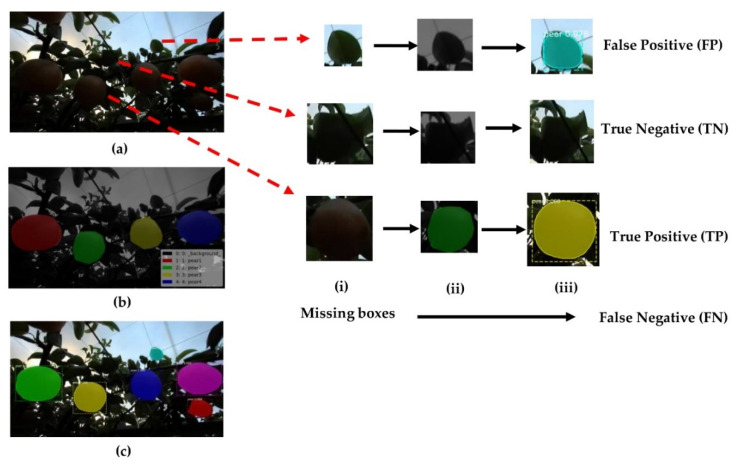
Pear prediction for determining *FP*, *TN*, *TP*, and *FN* using Mask R-CNN, (**a**) original image; (**b**) cv_mask input image before testing and (**c**) mask image after testing.

In this research, only pears needed to be detected, so pears were used as the only category. The task of Mask R-CNN was to detect the number of pears present in the pictures. Therefore, *TP* indicated the result that Mask R-CNN detected pears as pears in each image of the test set in the testing phase. *FN* pears were not identified. There was a missed identification: *TN* indicated a part that was not identified as a pear, and *FP* indicated that the background or the leaves were identified as a pear (Figure 7 and Figure 10).

Precision over reflected the proportion of correct classification in the number of positive samples classified by the model. Its expression is

(13) $$P\;=\;\frac{\mathrm{TP}}{\mathrm{TP}\;+\;\mathrm{FP}}$$

Recall was the ratio of the number of correct samples to the number of positive samples, and its expression is [32]

(14) $$R\;=\;\frac{\mathrm{TP}}{\mathrm{TP}\;+\;\mathrm{FN}}$$

Since there was only one category of fruit in this experiment, AP = mAP. Its value was between {0, 1}, and the closer to 1, the better the model recognition effect.

## 3. Results

### 3.1. Training Details

**Figure 11:**
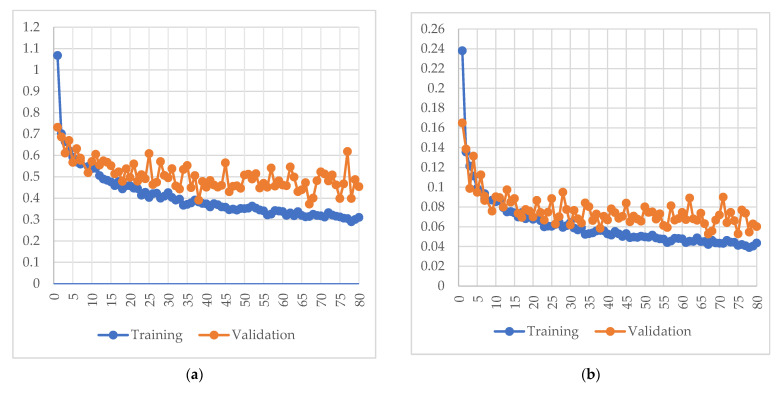

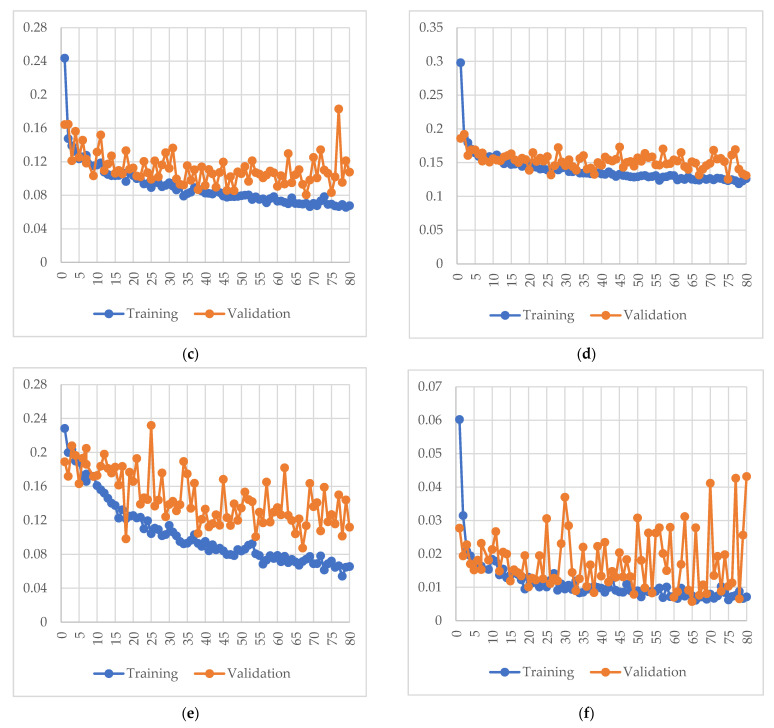
Mask R-CNN loss results from training losses and validation losses, (**a**) Total loss; (**b**) Mask R-CNN head bounding box loss; (**c**) Mask R-CNN head class loss; (**d**) Mask R-CNN mask loss; (**e**) RPN bounding box loss and (**f**) RPN class loss.

In this research, 9054 four-channel RGBA images (3018 images were original images and 6036 images were augmented images) in PNG format were used, and all images were taken by the same 3D stereo camera. The size of the validation set was adjusted by the loss function of the validation set. Initially, the training, validation, and testing sets were divided into a ratio of 6:3:1, that is, 5054 images for the training set, 2700 images for the validation set, and 900 images for the test set. The epoch was set to 80 with 500 steps in each epoch. During the experiment, since the Mask-RCNN could only use three-channel RGB images for the predicted images, the channels of the test set of 900 RBGA images were modified to RGB after the error was found. The following figures show the loss diagram of each partial function for this study (Figure 11a–f).

In this research, comparison experiments were conducted on the same datasets at different learning rates. From the training results, when the learning rate was set to 0.001, the training loss dropped to 0.3099 and the validation set loss dropped to 0.4637. Additionally, the Mask R-CNN head bounding box loss dropped to 0.0434 in the training set and the validation loss dropped to 0.0601 and the Mask R-CNN head class loss dropped to 0.0656 in the training set and the validation loss dropped to 0.1119; the Mask R-CNN mask loss dropped to 0.1260 in the training set and the validation loss dropped to 0.1310; the RPN bounding box loss dropped to 0.0677 in the training set and the validation loss was 0.1077; the RPN class loss in the training set was 0.0071 and the validation loss was 0.0432 (Figure 11a–f).

Figure 11a indicates the overall loss; by 80 epochs, each epoch was trained with 500 steps, which indicates that the model was good for this training. The Mask R-CNN bounding box loss denoted the loss of Mask R-CNN bounding box refinement, Mask R-CNN class loss denoted the head layer loss of classifier of Mask R-CNN, Mask R-CNN mask loss denoted the head layer mask binary cross-entropy loss of Mask, the RPN bounding box loss denoted the RPN bounding box loss, and the RPN class loss denoted anchor classifier loss.

Classification loss indicated how close the training model was to predicting the correct class. Mask R-CNN class loss was used as the head layer, and all objects were covered, while RPN class loss only covered the foreground and background of images. The border loss, on the other hand, responded to the distance between the real boxes and the predicted boxes. The Mask R-CNN mask loss responds to how close the model was to the predicted correct class mask. The sum of the above five losses constituted the overall loss.

### 3.2. Evaluation of Model Metrics

A series of weight files were obtained from the Mask R-CNN training and were used to evaluate the Mask R-CNN training model. The weight files left from the last training in the training process were selected to evaluate the test set.

The Precision (P), Recall (R), Average Precision (AP), and mean Average Precision (mAP) were used as the main parameters to evaluate the model in this research. We tested the different performances of the test set using the weights obtained from the training set after 80 epochs at different learning rates and the response plots of the Precision-Recall (PR) curves at a learning rate of 0.001. We also tested the same operation on the validation set with the weight trained by training sets, with overall mAP (IoU = 0.5). In addition, we tested different parts of the validation set, which was divided into three sections. One was original image datasets in the validation set, and another two were datasets after doing augmentation. We found that the results were nearly similar using the same weight tested in three sets: the original images (89.76%), rotation augmentation (84.47%), and flipped augmentation images (89.67%). However, while using all the datasets, including originals and two other augmented imageries in the validation process, the accuracy was increased (92.02%).

**Figure 12:**
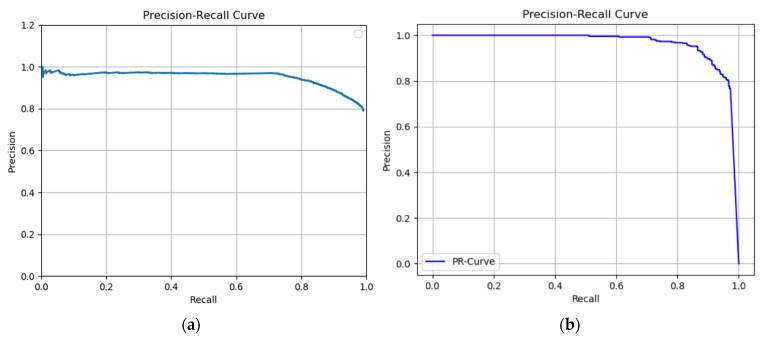
(**a**) Precision-recall curve of Faster R-CNN at learning rate = 0.001 in the testing set and (**b**) Precision-recall curve of Mask R-CNN at learning rate = 0.001 in the testing set.

Table 2 shows the comparison of mAPs in Mask R-CNN and Faster R-CNN. The Precision-recall curve of Faster R-CNN and Mask R-CNN from the testing set at different learning rates after 80 epochs (Figure 12).

### 3.3. Evaluation of Model Effectiveness

**Figure 13:**
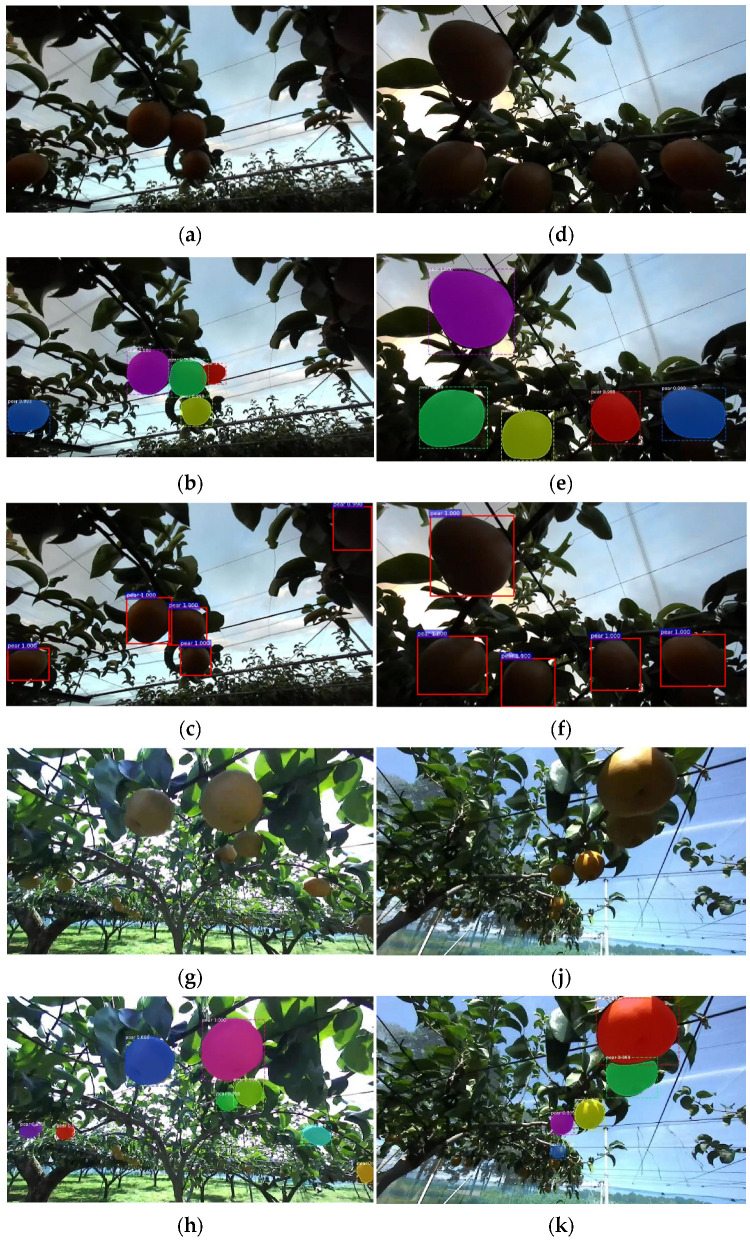

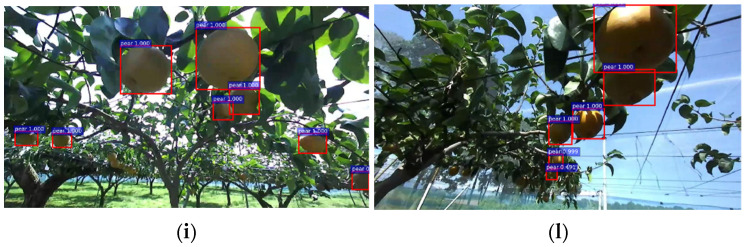
Results of Mask-RCNN in different situations. Recognition of (**a**–**c**): separated pears in low light; (**d**–**f**): aggregated pears in low light; (**g**–**i**): separated pears in strong light, and (**j**–**l**) aggregated pears in strong light. (**a**,**d**,**g**,**j**) Original image; (**b**,**e**,**h**,**k**) Testing image in Mask R-CNN; and (**c**,**f**,**i**,**l**) Testing image in Faster R-CNN.

**Figure 14:**
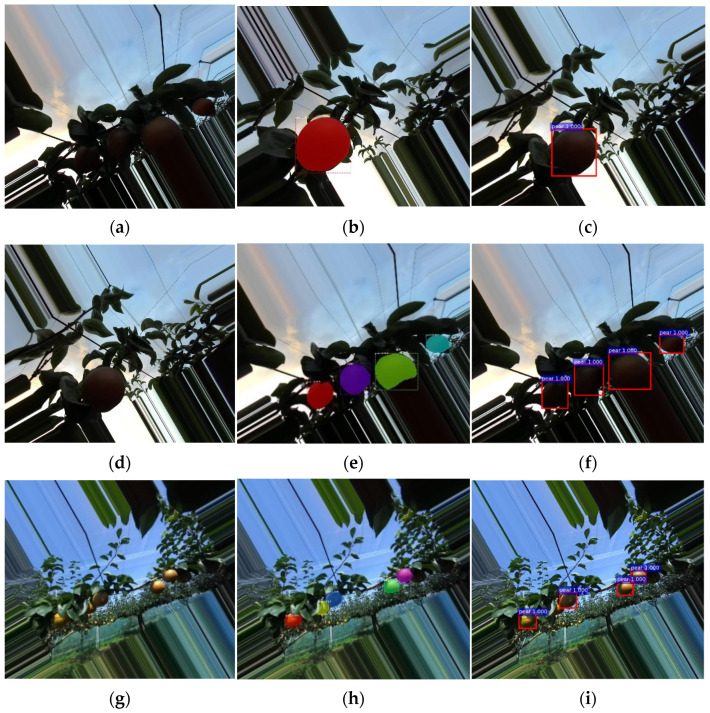

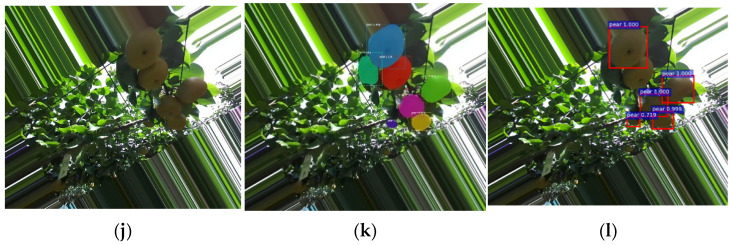
Results of Mask-R CNN in rotation angles. Recognition of (**a**–**c**): separated pear in low light; (**d**–**f**): aggregated pears in low light; (**g**–**i**): separated pears in strong light, and (**j**–**l**) aggregated pears in strong light (**a**,**d**,**g**,**j**) Original image; (**b**,**e**,**h**,**k**) Testing image in Mask R-CNN; and (**c**,**f**,**i**,**l**) Testing image in Faster R-CNN.

In this study, by creating a dataset and Mask R-CNN model using a 3D stereo camera, we found the best weights by comparing the fit of Mask R-CNN and Faster R-CNN with the same learning rate at lr = 0.001. By testing 900 images of the test set taken at different times, we obtained the following results by comparing the different effects of aggregating pears and separating pears under different illumination. Due to the problem of the light, branch, and leaf shading in the orchard, this research compared the test results of Mask R-CNN and Faster R-CNN under the light intensity from multiple pears in gathering and individual situations (Figure 13 and Figure 14).

The results showed that in the case of independent pear detection, the difference between the two was that Mask R-CNN generates both masks and bounding boxes, while Faster R-CNN detects pear-only generated bounding boxes. The detection accuracy of Mask R-CNN was significantly higher than that of Faster R-CNN under dark light conditions, and the accuracy of the bounding box of Mask R-CNN was higher than that of Faster R-CNN when pears were aggregated, or when the detection target is incomplete pears. Under bright light conditions, for the accuracy of independent pears, there was only a slight difference between the two. However, in the case of aggregated pears, Mask R-CNN had a higher correct recognition rate than Faster R-CNN.

We also tested the comparison of the recognition of pears in different situations for both after image rotation. When the pears were separated, the accuracy of the two only shows a small difference in the size of the borders. However, when the pears were aggregated, Faster R-CNN failed to recognize individual pears; Mask R-CNN had a higher recognition rate than Faster R-CNN in this case.

## 4. Discussion

Machine vision technology has become very popular due to the robust identification of objects and classification in industrial and agricultural technological applications. This approach can be implemented to future fruit picking robots or mechanical devices as machines replace human labor. Thus, the cost of population labor will be significantly reduced; in terms of detection, with the development of vision technology, the detection accuracy will also improve. In our study, we compared this technology with other deep learning models. The average precision of the YOLOv4 model for pear recognition in complex orchards was 93.76% and that of YOLOv4-tiny for pear recognition was 94.09% [32]. Moreover, by comparing the datasets using Faster R-CNN between apples, mangoes, and oranges, the mAP of apples was 86.87%, mangoes was 89.36%, and oranges was 87.39% [33]. The mAPs of this research were decreased when the camera field of view included fruit covered with leaves for the recognition of fruit with the Faster R-CNN algorithm. Even by labeling the fruit with different shading objects as different classes, mAPs for different classes did not significantly improve. The mAPs of leaf-occluded fruit, branch-occluded fruit, non-occluded fruit, and fruit-occluded fruit were 89.90%, 85.80%, 90.90%, and 84.80%, respectively [34]. By comparing the same dataset, which was taken by the same ZED camera, we knew that the mAPs of Faster R-CNN reached 87.52% on the testing set and 87.90% on the validation set. We also found that the performance of Faster-RCNN became less accurate when testing aggregated pears compared with Mask R-CNN. Therefore, Faster R-CNN achieved good results in detection and distinguished the types and individuals of objects while the mAPs of Faster R-CNN, in the case of recognizing one kind of fruit, were hardly a huge improvement even by data augmentation. Due to the complex environment of the orchard, for example, the color of the pears changed when the light intensity was different; because there were many branches, the pears and the leaves overlapped; or when multiple pears were clustered together, it was difficult to detect a single pear. Though Faster R-CNN had difficulties improving the accuracy of detection to a higher level and was prone to inaccurate detection results when testing aggregated pears, Mask R-CNN solved this problem perfectly. Mask R-CNN used instance segmentation technology and had advanced improvements in detecting aggregated pears and their accuracy. In this research, by using Mask R-CNN for the 3D stereo dataset, mAP (lr = 0.001) was 99.45% in the testing set and mAP(lr = 0.001) was 95.22%, which was much higher than that of Faster R-CNN. Due to this series of factors, Mask R-CNN has made great progress in detection. The mask generated by the Mask R-CNN network distinguished individual pears well when there were pear clusters in the environment.

We chose to use a 3D stereo camera for data acquisition. Traditional monocular cameras obtained better resolution for images; however, they had limitations in extended measurement. Although the principle of the monocular camera measurement method was simple, its accuracy was much lower than that of the 3D stereo camera. The ZED camera was different from ordinary traditional cameras; it obtained depth information by generating point clouds to calculate the actual distance between the camera and the pear. Using the ZED camera with Mask R-CNN combined instance segmentation and depth ranging to identify each of individual pears in complex orchards and calculate their actual distance. We also compared Faster R-CNN, which was a traditional visual recognition model that had an advantage in recognition speed, but Mask R-CNN had an advantage in accuracy. Furthermore, Mask R-CNN provided an increase in accuracy based on by adding a new mask branch. However, the disadvantage was also obvious: its detection speed was only 5 fps and there was a delay with the agricultural picking robots running at high speed. However, in slow picking, its effect was obviously remarkable at the development stages with high recognition accuracy. Since the ZED camera’s PERFORMANCE format was used, the resolution of the pictures it took was reduced compared to that of a regular camera. Therefore, while detecting, the dark leaves of shadows were detected as dark pears due to the influence of light. In this respect, the dataset accuracy needs to be improved.

However, in this study, the ranging module was not added, and only the ZED camera was used to make RGBA datasets. The ZED camera measured the distance through the depth point cloud. In future research, adding the comparison of the ranging module can further contribute to the development of agricultural robots for complex orchard work such as robotic picking machines using an end-effector or manipulator.

## 5. Conclusions

In this research, we developed a method for agricultural picking robots by using Mask R-CNN in an orchard from 3D stereo camera datasets. For the recognition of pears, the Mask R-CNN, had the mAPs of 95.22% for validation set and 99.45% was observed for the testing set. On the other hand, mAPs were observed 87.9% in the validation set and 87.52% in the testing set using Faster R-CNN. Furthermore, the 3D stereo camera was used with the Mask R-CNN dataset of pears in complex orchards for two different types of images: original RGBA and depth. Unlike the images taken by a conventional camera, the images taken by the 3D stereo camera could be used in the development of adding a distance measurement module and training a set of depth maps as a dataset in future work. Since we obtained the mask information of the identified pear, instead of obtaining the coordinates of the bounding boxes, so that the center point of the pears was calculated more accurately by the ZED camera, the accuracy of the coordinates of the center point obtained by masks was higher than the method of the center point obtained by the bounding boxes. This was because the positioning of the bounding boxes under different conditions showed some deviations. Therefore, we obtained high recognition accuracy on videos captured with the ZED camera, which was a 3D stereo camera, and organized them into applicable datasets using an advanced target detection model based on the Mask R-CNN with TensorFlow, including the Keras library. We used the ResNet101 and FPN as the backbone layer; And FCN was chosen as the head layer in Mask R-CNN to achieve higher accuracy in detection. The mAP of the dataset taken by the ZED stereo camera with an image size of 1280 × 720 using the Mask R-CNN at a learning rate of 0.001 reached 0.3099 using the training weight, and the mAP of the validation set reached 0.4637 at a learning rate of 0.001. We also verified the test speed and found that an image taken by the ZED camera had a test speed of 1.229 s on the Mask R-CNN because the average picking speed of some current picking robots is still about 6.000 s. The detection speed of Mask R-CNN could be seen as exploitable on a low-speed fruit picking mechanism but defective on a high-speed operational picking robot. Therefore, we provided another method to improve efficiency in agricultural production, thus solving the difficulty of previous vision models to recognize fruits because of their overlapping. Further research will be conducted to implement this vision recognition method for the development of agricultural robots in picking fruits using an end-effector or a manipulator.

## Figures and Tables

**Table 1:** Dataset collection times and light conditions in the complex orchard.

Date	Time	Light Condition
24 August 2021	9:00–10:00	High light
24 August 2021	18:00–19:00	Low light

**Table 2:** mAP results from 3D camera datasets using Mask R-CNN and Faster R-CNN in the testing set and validation set.

Model	Validation Set	Testing Set
Faster R-CNN	87.90%	87.52
Mask R-CNN	95.22%	99.45

## Data Availability

The dataset generated and analyzed during this study is available from the corresponding author upon reasonable request, but restrictions apply to the data reproducibility and commercially confident details.
